# Effect of Aqueous Extract and Polyphenol Fraction Derived from *Thymus atlanticus* Leaves on Acute Hyperlipidemia in the Syrian Golden Hamsters

**DOI:** 10.1155/2020/3282596

**Published:** 2020-03-28

**Authors:** Mhamed Ramchoun, Tarik Khouya, Hicham Harnafi, Souliman Amrani, Chakib Alem, Mohamed Benlyas, Fatima Kasbi Chadli, El-Hassane Nazih, Patrick Nguyen, Khadija Ouguerram

**Affiliations:** ^1^Laboratory of Biotechnology & Sustainable Development of Natural Resources, Polydisciplinary Faculty, University of Sultan Moulay Slimane, Beni Mellal 23000, Morocco; ^2^Biochemistry and Natural Substances Team, Department of Biology, Faculty of Sciences & Techniques, University of Moulay Ismail, Errachidia 52000, Morocco; ^3^Laboratory of Biochemistry, Department of Biology, Faculty of Sciences, University of Mohammed I, Oujda 60000, Morocco; ^4^INRA, UMR 1280, Physiology of Nutritional Adaptations, Nantes, France; ^5^CRNH, West Human Nutrition Research Center, CHU, Nantes, France; ^6^EA 2160 MMS Laboratory of Biochemistry, Faculty of Pharmacy, Nantes, France; ^7^ONIRIS, Nantes-Atlantic National College of Veterinary Medicine, UNE, Nantes, France

## Abstract

*Thymus atlanticus*, an endemic plant of Morocco, is traditionally used as a liniment or a drink to treat various diseases. However, there are few available scientific data regarding its biological effects. In this connection, the present study aimed to investigate the hypolipidemic and antioxidant effects of aqueous extract and polyphenol fraction of *Thymus atlanticus* in Syrian golden hamsters treated with Triton WR-1339 (triton, 20 mg/100 g body weight). The hamsters orally received the extracts (400 mg/kg), and blood samples were collected after 24 h of treatment to determine plasma lipid, insulin, and fasting blood glucose levels. Plasma malondialdehyde level and plasma total antioxidant (TAS) were also evaluated. The *T. atlanticus* extracts significantly decreased triglycerides, total cholesterol, VLDL-C, and LDL-C and increased HDL-C when compared with the hyperlipidemic group. Both extracts suppressed the effect of the triton injection on TAS and reduced the level of plasma malondialdehyde. The extracts produced no significant change in the blood glucose level but effectively prevented the mild hyperinsulinemia induced by triton. These findings suggest that *T. atlanticus* may be a useful alternative treatment for the control of hyperlipidemia and its related diseases.

## 1. Introduction

Hyperlipidemia is one of the major risk factors of atherosclerosis diseases [1]. It is defined as high plasma concentrations of total cholesterol (TC), triglycerides (TGs), and low-density lipoprotein cholesterol (LDL-C). Hyperlipidemia is associated with increased incidence of atherosclerosis, which is the main cause of cardiovascular diseases such as stroke, coronary artery disease, and peripheral artery disease [2]. Therefore, the reduction in the levels of plasma lipid is the main strategy for the control of the progressivity of cardiovascular diseases [3]. However, the current hypolipidemic drugs such as statins have many serious side effects [4]. Some plants have been used to reduce plasma levels of TC, TGs, and LDL-C in animal models [5, 6].

On the other hand, oxidative stress is involved in the pathogenesis of various diseases including atherosclerotic diseases [7]. The human body can generate numerous enzymatic and nonenzymatic antioxidants, but their amount could be insufficient to effectively neutralize the free radicals particularly under oxidative stress and inflammation conditions [8]. Hence, the supplementation of human diet with additional antioxidants derived from plants may have beneficial effects in atherosclerosis by preventing the accumulation of the free radicals and improving the antioxidant defense system [9].

In the traditional medicine, some subjects suffering from hyperlipidemia utilize herbal drinks as an alternative remedial tool to treat lipid metabolism disorders [10]. Moreover, the search for hypolipidemic drugs from medicinal plants has been intensified in recent years [11].

*Thymus atlanticus* (local Moroccan name: Z'itra or Azukni; English name: thyme) is an endemic medicinal herb of Morocco. Its leaves are used to prepare liniments or decoctions for treating several diseases including skin infections, rheumatism, and bronchitis and, generally, for its potentialities as an anti-inflammatory agent [10]. Moreover, it was reported that the aqueous extract of *T. atlanticus* possesses high total phenolic content and exhibited a potent anti-inflammatory effect in animal models of inflammation and inhibits blood coagulation *ex vivo and in vivo* [12, 13]. In addition, the aqueous extract of this plant showed potent *in vitro* antioxidant activity, but, to our knowledge, its antioxidant activity *in vivo* has not yet been studied [5, 13, 14]. A preliminary screening of some *Thymus* species from Morocco for hypolipidemic activity indicated that *T. atlanticus* showed possible hypolipidemic effects in a rat model [5]. However, in this latter study, the rats were treated with a high dose of aqueous extracts of the selected plants and the rat model is not an appropriate model for evaluating the hypolipidemic drugs because it has very low level of LDL-C and high level of high-density lipoprotein cholesterol (HDL-C) [15].

The hamster model is widely used for studying the lipid metabolism disorders and screening the new lipid-lowering drugs as the lipid metabolism of the hamster is similar to that of humans. Similar to humans, the hamster is endowed with cholesterol ester transfer protein (CETP) and all the enzymatic pathways of the metabolism of lipoproteins and bile [16, 17]. Therefore, this study was conducted to evaluate the effects of the aqueous extract and the polyphenol fraction of *T. atlanticus* on the plasma lipid concentrations and the antioxidant status in acute hypolipidemic hamsters.

## 2. Materials and Methods

### 2.1. Plant Material

Leaves of *T. atlanticus* (Ball) Roussine (synonym: *Thymus dreatensis*) were collected from the High Atlas Mountains (32° 15′ N, 5° 25′ E, 1995–2012 m), Errachidia, Morocco, in the month of April. The plant was authenticated by Dr. Ibn Tatou. A voucher specimen (no. RAB77496) was deposited in the herbarium of the Scientific Institute, Mohammed V University, Rabat, Morocco.

### 2.2. Preparation of Plant Extracts

The preparation of the aqueous extract (AE) was carried out in accordance with the method described in [18]. The extraction of polyphenol fraction (Pp) was performed based on the method of Jordan et al. [19].

### 2.3. Total Phenolic Content (TPC) Assay

The TPC assay was performed based on the Folin–Ciocalteu method [20] described in [18]. The TPC was expressed as mg caffeic acid equivalent (CAE) per g of dried extract.

### 2.4. *In Vitro* Antioxidant Activity

The 2,2 diphenyl picrylhydrazyl (DPPH) scavenging assay was performed based on the method described in [21] with minor modifications [18]. Antioxidant capacity was evaluated as the capacity of extracts to reduce Fe^3+^ to Fe^2+^ by the Benzie and Strain method [22]. The inhibition of *β*-carotene-linoleic acid bleaching by *T. atlanticus* extracts was evaluated according to Elzaawely et al. [23].

### 2.5. Animals and Experimental Design

Animal procedures were carried out in strict accordance with the recommendations in the Guide for the Care and Use of Laboratory Animals of the European Union “European directive 2010/63/EU” and of the Ethical Principles of Animal Experimentation of the Ministry for Food, Agriculture, and Fisheries (France). The protocol was approved by the local committee (Bretagne-Pays de la Loire Committee). All surgery were performed under sodium pentobarbital anesthesia, and all efforts were made to minimize suffering.

Forty adult male golden Syrian hamsters (70–90 g, 8weeks old, Animal Experimental Station, Nantes, France) were used as experimental animals in the current study. They were maintained in standard cages (3 hamsters per cage) under a 12 h light/dark cycle at a temperature of 22 ± 3°C. The experimental hamsters were divided into four groups of 10 animals each. Hamsters were rendered hyperlipidemic by an intraperitoneal injection of Triton WR-1339 (triton, 20 mg/100 g BW) one hour after the administration by oral route of distilled water (hyperlipidemic group, HG), AE, or Pp at a dose of 400 mg/kg BW. The control (normolipidemic group, NG) was not injected with triton and received distilled water only. After 24 h of treatment, blood was taken from the retro-orbital sinus, after a slight anesthesia with isoflurane. The blood samples were centrifuged (10 min, 4°C, 3000 g), and the obtained plasma was used for the analysis of glucose, insulin, and lipids. The hamsters were euthanized at the end of the experiment with an intraperitoneal injection of sodium pentobarbital (60 mg/kg BW).

#### 2.5.1. Dosage of Plasma Total Cholesterol (TC) and Triglycerides (TGs)

The TC and TGs in plasma were quantified using enzymatic kits (Bio-Merieux, France), according to the manufacturer's instructions.

#### 2.5.2. FPLC Analysis

The cholesterol and TG profiles in lipoproteins were determined using fast protein liquid chromatography (FPLC) (AKTA FPLC SYSTEM, GE Healthcare, USA). The detailed procedure is available in a previous publication [24].

#### 2.5.3. Glucose and Insulin Measurement

The blood glucose level was measured in a drop of blood immediately after sampling using a glucometer (Roche Diagnostics, France). Insulin was analyzed in plasma prepared from the blood collected via the retro-orbital sinus of food-deprived hamsters using the rat insulin ELISA kit (Shibayagi, Japan).

#### 2.5.4. *In Vivo* Antioxidant Activities

The level of malondialdehyde (MDA) in plasma was measured based on the method described in [24]. Plasma total antioxidant status (TAS) was determined according to the manufacturer's protocol of the rat TAS reagent kit (TAS, RANDOX Laboratories Ltd, United Kingdom).

### 2.6. Statistical Analysis

Data were represented as mean ± standard deviation (SD) (*n* = 10). Statistical analysis was performed using Statview software (SAS Institute Inc., 100 SAS Campus Drive, Cary, NC 27513-2414, USA). All comparisons between groups were made by means of one-way ANOVA test followed by post hoc analysis (Tukey's or Dunnett's test). Differences were statistically significant at *p* < 0.05.

## 3. Results

### 3.1. Total Phenolic Content of *T. atlanticus* Extracts

The yield of extraction of AE and Pp was 15% (w/w) and 9 % (w/w), respectively. The AE and Pp had the TPC values of 481.1 ± 23.42 and 589.34 ± 43.56 mg CAE/g of dried extract, respectively (Table 1).

### 3.2. *In Vitro* Antioxidant Activity

The *in vitro* antioxidant activity of AE and Pp was evaluated using three different assays.

Results are presented as mean ± SD, *n* = 6. ^†^Total phenolic content is expressed as mg caffeic acid equivalent per g of dried extract.

The results are presented in Table 2. The IC_50_ values of AE and Pp in DPPH scavenging assay were 0.44 ± 0.02 mg/mL and 0.012 ± 0.005 mg/mL, respectively. Trolox had the IC_50_ value of 0.51 ± 0.01 mg/mL. The DPPH scavenging activity of Pp and AE was significantly higher than that of Trolox (*p* < 0.05).

The FRAP values of AE and Pp were 50.31 ± 1.65 and 67.96 ± 4.12 mmol Trolox/g of dried extract, respectively (Table 2). The *β*-carotene bleaching results of AE and Pp present the IC_50_ values of 46.8 ± 4.15 and 13.05 ± 1.35 mg/mL, respectively. BHT had an IC_50_ value of 0.12 ± 0.03 mg/mL.

### 3.3. Effects of AE and Pp on Hyperlipidemia in Hamsters

#### 3.3.1. Plasma TC and TGs

**Figure 1:**
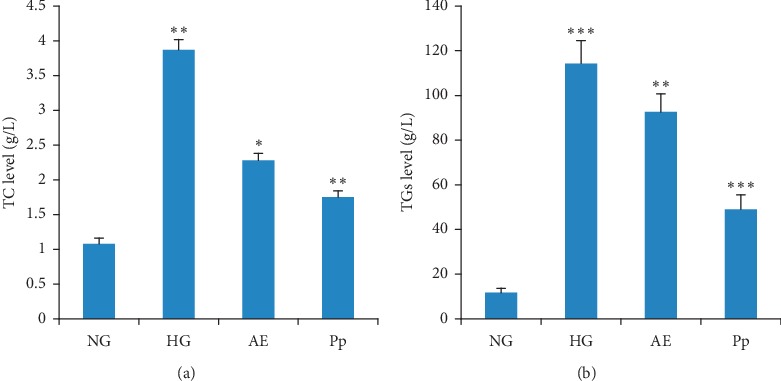
Effect of AE and Pp of *Thymus atlanticus* on lipid concentrations. (a) TC. (b) TGs. AE, aqueous extract-treated hamsters; HG, hyperlipidemic hamsters; NG, normolipidemic hamsters; Pp, polyphenol fraction-treated hamsters; TC, total cholesterol; TGs, triglycerides. Results are expressed as mean ± SD and analyzed with ANOVA followed by Tukey's test, *n* = 10. ^*∗*^*p* < 0.05, ^*∗∗*^*p* < 0.01, and ^*∗∗∗*^*p* < 0.001; HG *versus* NG, AE, and Pp *versus* HG.

Figure 1 summarizes the level of plasma TC and TGs. The injection of triton resulted in a significant increase in the level of plasma TC (72%; *p* < 0.01) and TGs (89.8%; *p* < 0.001) in HG when compared with hamsters not treated with triton (NG).

The plasma TC and TGs registered a significant decline in the AE-treated group by about 41% (*p* < 0.05) and 19.03% (*p* < 0.01), respectively, when compared with HG (Figure 1(a)). The TC and TG levels were also significantly decreased in the Pp-treated group by about 55% (*p* < 0.01) and 57% (*p* < 0.001), respectively (Figure 1(b)).

The plasma TC and TGs registered a significant decline in the AE-treated group by about 41% (*p* < 0.05) and 19.03% (*p* < 0.01), respectively, when compared with HG (Figure 1(a)). The TC and TG levels were also significantly decreased in the Pp-treated group by about 55% (*p* < 0.01) and 57% (*p* < 0.001), respectively (Figure 1(b)).

#### 3.3.2. FPLC Profiles of TC and TGs in VLDL, LDL, and HDL

**Figure 2:**
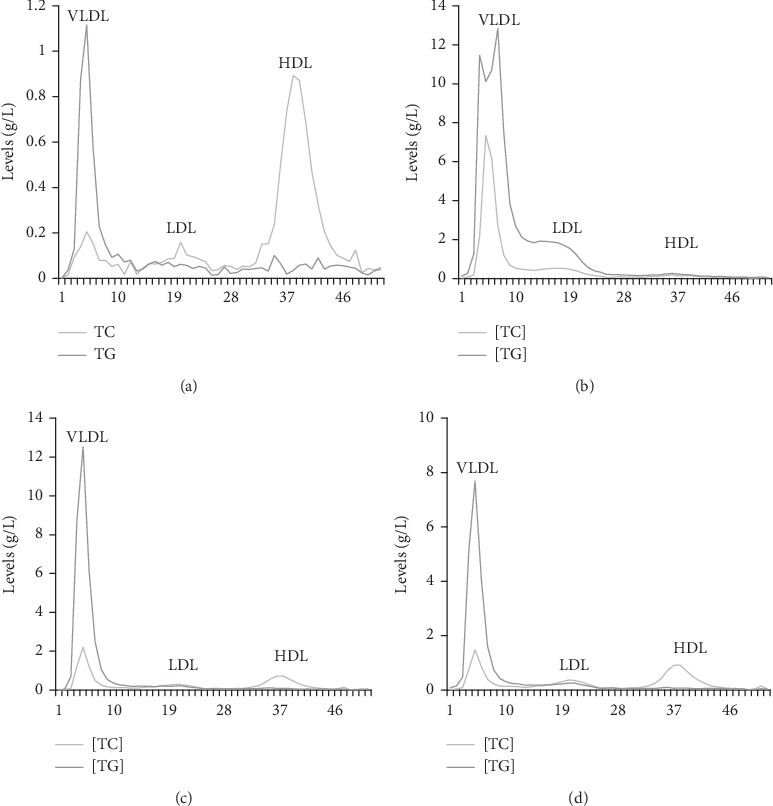
Lipoprotein profiles determined by FPLC. (a) Normolipidemic group. (b) Hyperlipidemic group. (c) AE-treated hamsters. (d) Pp-treated hamsters. HDL, high-density lipoprotein; LDL, low-density lipoprotein; TC, total cholesterol; TG,triglycerides; VLDL, very LDL.

The plasma lipoprotein profiles obtained by the FLPC analysis are shown in Figure 2. The results showed that the intraperitoneal injection of triton significantly increased the levels of VLDL-TGs and VLDL-C by about 92% and 97%, respectively, and effectively decreased HDL-C by about 84%, in comparison with the NG (Figures 2(a) and 2(b)).

The levels of VLDL-TGs and VLDL-C were seen to be significantly decreased in the AE-treated group by about 2% and 72% and in the Pp-treated group by 40% and 80%, respectively (Figures 2(c) and 2(d)). In addition, AE and Pp significantly increased HDL-C by about 88% and 90%, respectively (Figures 2(c) and 2(d)). Plasma LDL-C and LDL-TGs concentrations were very low (almost zero g/L) in AE-treated and Pp-treated groups (Figures 2(c) and 2(d)).

#### 3.3.3. Plasma Insulin and Blood Glucose

**Figure 3:**
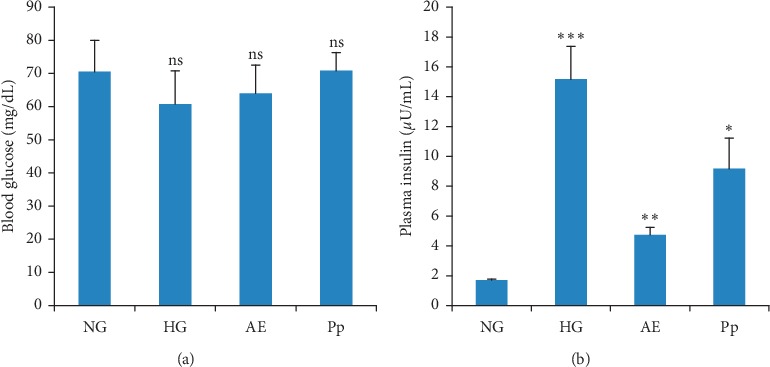
Levels of blood glucose (a) and plasma insulin (b). AE, aqueous extract-treated hamsters; HG, hyperlipidemic group; NG, normolipidemic group; Pp, polyphenol fraction-treated hamsters. Results are presented as mean ± SD and analyzed with ANOVA followed by Tukey's test, *n* = 10. ^*∗*^*p* < 0.05, ^*∗∗*^*p* < 0.01, and ^*∗∗∗*^*p* < 0.001; ns, not significant; HG *versus* NG, AE, and Pp *versus* HG.

There were no significant differences in blood glucose levels between the groups (Figure 3(a)). The injection of triton resulted in a significant (*p* < 0.001) increase of plasma insulin level by about 89% when compared with theNG. The levels of plasma insulin were significantly decreased by about 69% (*p* < 0.01) and 40% (*p* < 0.05) in AE and Pp groups, respectively, when compared with the NG (Figure 3(b)).

#### 3.3.4. *In Vivo* Antioxidant Activity

**Figure 4:**
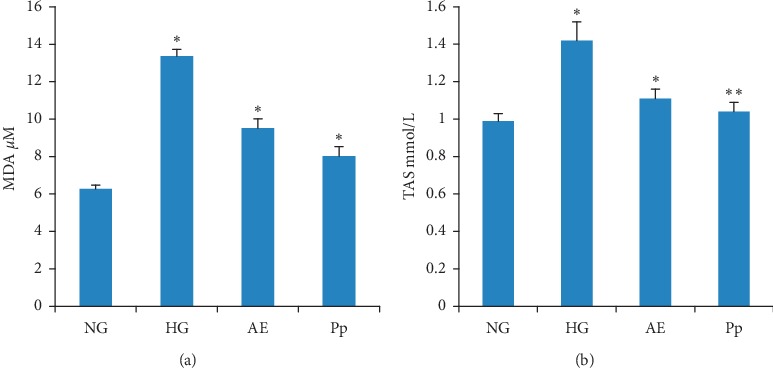
Plasma malondialdehyde levels (a) and plasma total antioxidant status (TAS) (b). AE, aqueous extract-treated hamsters; HG, hyperlipidemic hamster; NG, normolipidemic hamsters; Pp, polyphenol fraction-treated hamsters. Results are presented as mean ± SD and analyzed with ANOVA and Tukey's test, *n* = 10. ^*∗*^*p* < 0.01 and ^*∗∗*^*p* < 0.001; HG *versus* NG, AE, and Pp *versus* HG.

Figure 4 shows the plasma level of MDA Figure 4(a) and the plasma TAS Figure 4(b). The results indicate that the MDA level significantly increased by about 53% (*p* < 0.001) in the HG when compared with the NG. The AE and Pp significantly (*p* < 0.001) decreased the MDA level by about 29% and 40%, respectively, when compared with the HG (Figure 4(a)).

The plasma level of TAS was significantly increased in the HG when compared with the NG (1.42 ± 0.04 mmol/L vs. 0.99 ± 0.05 mmol/L; *p* < 0.01) (Figure 4(b)). The *T. atlanticus* extracts effectively suppressed the effect of triton on TAS. The plasma level of TAS in AE-treated and Pp-treated groups was similar to that in theNG (*p* > 0.05).

## 4. Discussion

In the present study, hamsters were rendered hyperlipidemic by an intraperitoneal injection of triton to evaluate the hypolipidemic and antioxidant effects of two polyphenol-rich extracts of *T. atlanticus*. The hamster is the best model to study the lipid metabolism disorders and for screening new lipid-lowering drugs as the lipid metabolism of hamsters is similar to that of humans. Like humans, the hamster is endowed with cholesterol ester transfer protein (CETP) and all the enzymatic pathways in the metabolism of lipoproteins and bile [16, 17]. In addition, triton is known to cause hyperlipidemia through several mechanisms, mainly by inhibiting lipoprotein lipase activity, which can result in the blockage of TG-rich lipoproteins clearance [25].

In the present study, administration of a single dose of AE or Pp of *T. atlanticus* ameliorated acute hyperlipidemia in hamsters, evidenced by a significant decrease in the levels of plasma TC and TGs. We also found that the *T. atlanticus* extracts suppressed the effect of triton on antioxidant status and effectively prevented the increase of plasma MDA. In addition, these extracts also prevented the changes induced by triton in lipoprotein profiles. Moreover, the injection of triton produced a marked increase in VLDL-TGs and LDL-C levels and a significant decrease in the HDL-C level.

Our results are in accordance with the results of Ramchoun et al. (2012) who have reported that the crude AE of *T. atlanticus* prevented the increase of plasma TC, TGs, and LDL-C and effectively increased the HDL-C level in the rat model of acute hyperlipidemia. The effects of *T. atlanticus* extracts on lipid profiles in rat were similar to those of fibrates (fenofibrate 65 mg/kg BW), which are one of the major classes of the current hypolipidemic drugs, mainly used to reduce plasma TGs concentration [5].

Elevated plasma levels of TC, LDL-C, and TGs are the major risk factors for heart disease and stroke in the world [26]. The reduction of circulating levels of these particles result in a significant reduction in the incidence and the severity of heart diseases [3]. Therefore, the hypolipidemic drugs such as statins and fibrates are the main strategy for controlling and preventing cardiovascular diseases [4, 27]. However, the statins and fibrates have many side effects. This has attracted a great attention for the discovery of new hypolipidemic drugs with fewer side effects [4, 28]. In this context, some extracts derived from medicinal plants have been found to reduce atherosclerotic lesions and attenuate atherosclerosis development, along with a reduction of plasma lipid concentrations (TC, TGs, and LDL-C) and atherogenic indexes, in animal models for atherosclerosis [29, 30]. In the present study, the AE and Pp of *T. atlanticus* effectively reduced TC, LDL-C, and TGs levels, which suggests beneficial effects of these extracts in the control of cardiovascular diseases. On the other hand, the plasma HDL-C level is inversely associated with the prevalence of heart diseases [31]. We found that the HDL-C level was considerably increased upon treatment with a single dose of AE or Pp. The atherogenic effects of HDL-C are due to its role in reverse cholesterol transport and to its associated antioxidant enzymes such as paraoxonase 1 (PON1), which can degrade various proatherogenic compounds resulted from lipid oxidation [32]. Among these oxidation products, MDA, a marker of lipid oxidation and cardiovascular disease, is a toxic compound that can interact with DNA and proteins, which results in the dysfunction of the endothelium and other atherogenic events [33]. The reduction of plasma level of MDA in the hyperlipidemic hamsters receiving the *T. atlanticus* extracts suggests a preventive effect of these extracts against lipid oxidation and atherosclerosis development. Like hyperlipidemia, oxidative stress is a serious risk factor for many diseases such as heart diseases [34]. The lipid oxidation results in the generation of other atherogenic products such as oxidized LDL-C, which can induce endothelium dysfunction and activate inflammation and coagulation systems, leading to the development and progression of the atherosclerotic plaque [35]. In the present study, the *T. atlanticus* extracts suppressed the effect of triton on TAS and showed potent antioxidant activity, evidenced by a greater capacity to donate electrons (FRAP assay) and scavenge free radicals (DPPH assay) in comparison with standard antioxidant, Trolox. These findings are in accordance with our previous studies, which have demonstrated that the *T. atlanticus* extracts protect the blood red cell membranes against 2,2′-azobis (2-amidinopropane) dihydrochloride-mediated oxidation and plasma lipids against CuSO_2_-mediated oxidation [13]. Most studies have confirmed that the antioxidants can prevent lipid oxidation mediated by the free radicals and alleviate atherosclerosis and its related diseases [36]. The antioxidant proprieties of the plant are due to its secondary metabolites, mainly polyphenols [37]. Moreover, *Thymus* species have been reported to have high amount of phenolic acids and flavonoids [18, 38]. Based on previous phytochemical analyses [5, 12, 13], the HPLC chromatograms of the *T. atlanticus* AE and Pp showed the presence of various phenolic compounds with predominance of rosmarinic acid, caffeic acid, and quercetin. As mentioned previously [13], *T. atlanticus* showed highest total phenolic and flavonoid contents than other *Thymus* species.

Concerning the mechanism of the hypolipidemic effects of the *T. atlanticus* extracts, at this time, we did not examine further on this issue. It has been reported that polyphenols inhibit the activity of 3-hydroxy-3-methyl-glutaryl-CoA reductase *in vitro* [39], reduce intestinal cholesterol absorption [40], and inhibit the activity of CETP [41]. Moreover, the pharmacological inhibition of CETP has become a strategy for increasing HDL-C and decreasing LDL-C levels and has proven to be effective in the treatment of atherosclerosis and cardiovascular diseases [42]. The effect of *T. atlanticus* on decreasing LDL-C and increasing HDL-C may be partly due to the inhibition of CETP. Furthermore, the simultaneous decline in TC and LDL-C in plant-treated groups upon extract administration allows us to suggest that the *T. atlanticus* extracts decreased TC by enhancing the elimination of the LDL-C particles through their hepatic receptors.

Epidemiologic studies have shown that the intake of dietary polyphenols could regulate plasma lipid concentrations and decrease the prevalence of cardiovascular diseases [43, 44]. Polyphenols have beneficial effects on the regulation of the PNO1 expression and the stabilization of lipoproteins [45]. In addition, polyphenols decrease oxidative stress in macrophages and enhance their efficacy in cholesterol efflux, ameliorate antioxidant status in atherosclerotic lesions, and attenuate atherosclerotic plaque progression [43,44]. Atherosclerosis is a multifactorial process that implicates many systems such as inflammation and coagulation. Thus, the antiatherogenic effect of polyphenols could also be due to the inhibition of proinflammatory enzymes, cytokines secretion, and coagulation factors [9]. In this connection, a previous study has reported that the *T. atlanticus*' AE suppressed inflammation induced by carrageenan and croton oil in animal models and effectively inhibited blood coagulation *in vitro* [13]. This suggests additional therapeutic effects of the active compounds of *T. atlanticus* in cardiovascular diseases.

In addition, the injection of triton significantly increased the plasma insulin level without affecting the blood glucose level in hamsters. Both AE and Pp produced no significant change in the glucose level, but they effectively suppressed the effect of triton on the insulin level and prevented against mild hyperinsulinemia. Diabetes is associated with lipid level abnormalities and accelerates atherosclerotic cardiovascular diseases [46]. Moreover, insulin regulates lipid metabolism at several levels such as the expression and the synthesis of lipoprotein lipase and the synthesis and the clearance of VLDL and chylomicrons [47]. The increase in plasma lipids after the injection of triton may be due to the increase in plasma insulin in hamsters. In fact, the beneficial effect of the *T. atlanticus* extracts on lipid metabolism may be partly attributed to their action on the insulin level. Clinical trials have been reported that the intake of polyphenols alleviates cardiovascular risk in people with diabetes [48]. Most studies verified that the benefits of rosmarinic acid, which was the most abundant phenolic compound in *T. atlanticus*[13], are partly due to its antidiabetic effects. Rosmarinic acid has been found to improve insulin sensitivity and glucose uptake in cell culture and in rodents. It exhibited potent inhibitory effect on *α*-glucosidase and *α*-amylase [49]. The inhibition of these enzymes is widely used as a strategy to manage hyperglycemia associated with type 2 diabetes [50].

A recent study has demonstrated that the aqueous extract of *T. atlanticus* can be classified as a low-toxicity extract according to the Organization for Economic Co-operation and Development [12].

There are few limitations in this study that could be addressed in future works. Chronic hyperlipidemia induced by high-fat diet was not studied because we focused only on acute hyperlipidemia and the mechanisms of hypolipidemic action of *T. atlanticus* have not been explored.

## 5. Conclusion

Oral administration of the *T. atlanticus* extracts ameliorates the lipidemic state in triton-induced hyperlipidemic hamsters via decreasing plasma cholesterol and LDL-C levels, increasing HDL-C, improving antioxidant status, and preventing mild hyperinsulinemia. The active compounds in the *T. atlanticus* extracts could be used in the treatment of hyperlipidemia.

## Figures and Tables

**Table 1:** Total phenolic content of the aqueous extract and the polyphenol fraction.

	Aqueous extract	Polyphenol fraction
Total phenolic content^†^	481.10 ± 23.42	589.34 ± 43.56

**Table 2:** Antioxidant activity (DPPH, FRAP, and *β*-carotene assay) of the aqueous extract and polyphenol fraction.

Extracts	DPPH (IC_50_ mg/mL)	FRAP (mmol Trolox/g DR)	*β*-carotene (IC_50_ mg/mL)
Aqueous extract	0.44 ± 0.02^*∗*^	50.31 ± 1.65^ns^	46.8 ± 4.15^*∗∗∗*^
Polyphenol fraction	0.012 ± 0.005^*∗∗∗*^	67.96 ± 4.12^*∗∗*^	13.05 ± 1.35^*∗∗∗*^
Trolox	0.51 ± 0.01	—	—
BHT	—	—	0.21 ± 0.03

BHT, butylated hydroxytoluene; DPPH, DPPH scavenging activity; DR, dry extract; FRAP, ferric reducing antioxidant power. Results are presented as mean ± SD and analyzed with ANOVA and Dunnett's test, *n* = 6. ^*∗*^*p* < 0.05, ^*∗∗*^*p* < 0.01, and ^*∗∗∗*^*p* < 0.001; ns, no significance *versus* Trolox or BHT.

## Data Availability

The data used to support the findings of this study are available from the corresponding author upon request.
